# Supplementary material for: Predicting visual function by interpreting a neuronal wiring diagram
Source: Nature. 2024 Oct 2;634(8032):113–23. doi: 10.1038/s41586-024-07953-5 (PMC11446822; doi:10.1038/s41586-024-07953-5)
Supplement: Supplementary file 6 — CRF and ERF predictions for individual TmY4 and TmY9 cells. Analogous to Supplementary Data 3, but for TmY target types. Shown are the top four monosynaptic pathways, the strongest pathway passing through each of the top ten intermediary types (ranking from Extended Data Fig. 7), and the trisynaptic pathway Tm1–TmY–Dm3–TmY (see the section entitled Prediction of spatial normalization). [file 41586_2024_7953_MOESM6_ESM.zip › DataS4/TmY9qΓèÑ/720575940623011549.pdf]

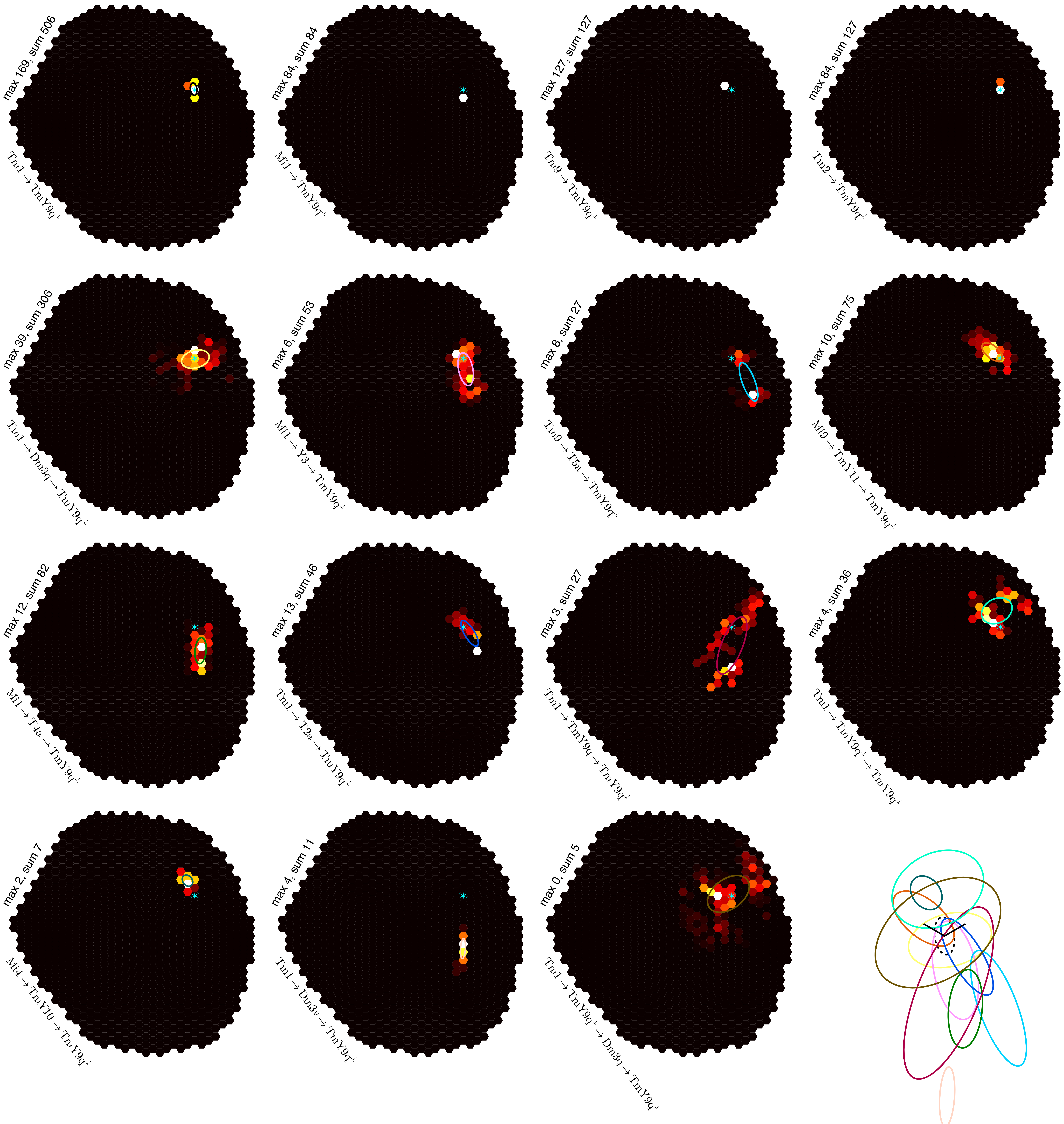
max 169, sum 506
Tm1 → TmY9q⊥
max 84, sum 84
Mi1 → TmY9q⊥
max 127, sum 127
Tm9 → TmY9q⊥
max 84, sum 127
Tm2 → TmY9q⊥
max 39, sum 306
Tm1 → Dm3q → TmY9q⊥
max 6, sum 53
Mi1 → Y3 → TmY9q⊥
max 8, sum 27
Tm9 → T5a → TmY9q⊥
max 10, sum 75
Mi9 → TmY11 → TmY9q⊥
max 12, sum 82
Mi1 → T4a → TmY9q⊥
max 13, sum 46
Tm1 → T2a → TmY9q⊥
max 3, sum 27
Tm1 → TmY9q → TmY9q⊥
max 4, sum 36
Tm1 → TmY9q⊥ → TmY9q⊥
max 2, sum 7
Mi4 → TmY10 → TmY9q⊥
max 4, sum 11
Tm1 → Dm3v → TmY9q⊥
max 0, sum 5
Tm1 → TmY9q⊥ → Dm3q → TmY9q⊥
